# Exploring the potential role of community engagement in evaluating clinical and translational science grant proposals

**DOI:** 10.1017/cts.2018.311

**Published:** 2018-09-18

**Authors:** Jeffrey W. Treem, Margaret Schneider, Robynn L. Zender, Dara H. Sorkin

**Affiliations:** 1 Department of Communication Studies, The University of Texas at Austin; 2 Institute for Clinical and Translational Science, University of California, Irvine

**Keywords:** Community engagement, grants, evaluation, scientific review, scientific merit

## Abstract

**Introduction:**

This study explored the effects of integrating community members into the evaluation of clinical and translational science grants.

**Methods:**

The University of California, Irvine Institute for Clinical and Translational Sciences (ICTS) engaged 21 community reviewers alongside scientific reviewers in a 2-stage process of evaluating research proposals. In Stage 1 reviewers scored proposals, and during Stage 2 two study sections convened: one a mix of community reviewers and scientific reviewers, and one only engaging scientific reviewers. In total, 4 studies were discussed by both study sections.

**Results:**

Comparisons of reviews revealed little difference between ratings of community reviewers and those of scientific reviewers, and that community reviewers largely refrained from critiquing scientific or technical aspects of proposals.

**Conclusions:**

The findings suggest that involving community reviewers early in the grant cycle, and exposing them to the entirety of the review process, can bolster community engagement without compromising the rigor of grant evaluations.

## Introduction

Community engagement has been recognized as a fundamental element of clinical and translational science and a specific emphasis for NIH funding to the Clinical and Translational Science Awards (CTSA) [1]. Because community members can provide insights from work and experience outside of traditional academic or research environments they are critical to scientific translation, which is defined as “the process of turning observations in the laboratory, clinic and community into interventions that improve the health of individuals and the public (https://ncats.nih.gov/translation/spectrum).” However, despite consensus regarding the value of community engagement for clinical and translational science initiatives, there is little clarity on the extent and the process by which the community should be embedded in the translational cycle [2].

One reason that community engagement is difficult to effectively integrate into translational science is that it requires interactions with stakeholders (e.g., community leaders, industry) and resources (e.g., legal counsel, product development) that are not inherently involved, or incentivized, in the processes of institutional research. The novel demands and diversity of this type of engagement requires a special skill set for health researchers, and choosing to embark on community-engaged research (CEnR) changes academicians’ scholarly trajectory substantially. CEnR requires significant time commitments, relationship and trust-building, and perseverance that typically do not lead to quick or numerous publications or grant proposals, so understanding the benefit to scientists of engaging the public in research is critical [3].

Likewise, community stakeholders can be intimidated by or unknowledgeable about research methods, processes, and researchers, and may not understand how engaging in research might inform activities of their organizations, or patient knowledge and behavior [4]. Further, how well each partner in CEnR understands the others’ needs, expectations, and capacity for partnered work impacts the outcomes of studies and longevity of partnerships [5]. Therefore, integrating community engagement into clinical and translational science is both structurally difficult in the sense that institutional researchers may lack existing relationships with community stakeholders, and functionally problematic in that the time and resources required to build the requisite relationships may hinder the short-term productivity of research efforts.

Models of effective community engagement aim to conceptualize the components and dynamics of successful collaborations between scientists and community stakeholders in each phase of the translational research spectrum, from first tests in humans (i.e., T1) through to the dissemination of new treatments to the public (i.e., T4). Community engagement is posited to engender better quality research, expand the applicability of research, empower patients, improve dissemination and uptake of results, and respond to a moral imperative to include a broad community of stakeholders in research processes [6, 7]. Typically, however, patients, the public, and advocacy stakeholders are neither conceptualized to be engaged, nor actually engaged, in the early phases of the translational continuum [8, 9]. To date, the majority of community engagement efforts have focused on informing patient-centered health initiatives, recruiting and soliciting participation in clinical studies or interventions, or facilitating the dissemination of health information [10–12]. In each of these contexts the role of the community is seen as supplementing and complementing the work of scientists and researchers. Individuals and groups are engaged to provide knowledge, insights, and expertise regarding the community itself, and the needs of its members. Though community engagement is a core aspect of the CTSA program, it emerges largely in the T2 (from bedside to clinical practice) to T4 (from health care delivery to community, public health, and public policy) phases of translational science.

Less common are efforts to engage community members in decisions regarding investments in scientific and research initiatives. Despite the fact that participation of community members in the peer review process is part of The National Institutes of Health Director’s Council of Public Representatives’ framework for community engagement [13], this approach is rarely implemented. A survey of 49 CTSA hubs, conducted in 2016, found that 31% of the respondent institutions involved community members in their pilot grant review process [14], yet little is known about how the community input impacts the evaluation process. There have been some reports on the process behind efforts to include community members in the review of grants for translational science. For example, the Michigan Institute for Clinical and Health Research (MICHR) has piloted and described a process in which community members were involved in the scoring of pilot grants to support community-academic partnerships [15]. Another description of the process of involving nonscientific reviewers in evaluating funding proposals is represented by the work done by the Patient-Centered Outcomes Research Institute (PCORI) [16] to include practitioners and stakeholders in the peer review of patient-centered study proposals. This research found that the evaluations of nonscientific researchers initially differed from those of scientists, but that the scores converged after in-person discussion sessions. Participation of nonscientific reviewers also changed the ranking of proposals, as opposed to when scientists alone scored proposals. The researchers recommended further study of the potential role of nonscientific reviewers in the peer review process.

These cases reflect a belief that community members possess some unique or meaningful knowledge regarding the needs of a particular population, group, or community. In the case of PCORI, the community members represent the “voice of the patient and/or stakeholder” and are viewed as advocates who can speak to the clinical experience of those receiving medical care. Similarly, the relevance of community members in informing funding decisions may be more apparent in situations where the work is designed to have an immediate clinical or public-facing implementation; that is, in the later phases of the translational research spectrum: T3 and T4. Although NCATS has called for community engagement across the spectrum of research from basic science and first-in-human studies (T0-T1) to community and population health research (T4), only a handful of examples of T0–T1 community engagement exist in the literature [17].

This investigation extends our understanding of the potential role of community members in the evaluation of CTSA funding opportunities by looking at the inclusion of the community members in the evaluation of pilot clinical and translational science research projects that span the entire translational spectrum. There are both theoretical and practical reasons to examine the engagement of community across all phases of the translational cycles. Including community reviewers in the peer review process tests the assumption that scientific reviewers exclusively possess the expertise to evaluate research proposals. This issue is increasingly relevant given that clinical and translational science research teams are often interdisciplinary, which may create difficulties in evaluation across areas of expertise. Different domains of practice possess different terms, instrumentation, and norms of publication that could influence assessments of grant applications. Additionally, the difficulties of recruiting, assigning, and cajoling reviewers creates inherent inconsistencies between the level of expertise a scientific reviewer may have related to the proposal they evaluate. Finally, though the goal of peer review of proposals is to evaluate the merit of research projects, what is materially assessed by reviewers is not the actual research, but rather the proposal document. This creates the possibility of a mismatch between the merit, significance, and value of the scientific project proposed, and the ability of individuals to communicate those strengths effectively to reviewers. Examining the review process in more detail will help reveal what factors influence reviewers’ evaluations and the consequences for funding decisions.

Investigating the potential role of community members in the evaluation of CTSA funding opportunities presents 2 clear benefits. First, it offers an empirical analysis of whether the assessments of proposals differ among these groups, and if so, in what ways. This benefit includes enhancing both the understanding of the potential consequences that including community reviewer evaluations might have in the scoring of proposals and an understanding of what factors influence the respective scores provided. Second, this project may provide additional insights into the potential value of the inclusion of community members at this stage of clinical and translational science. Our examination of the potential role of community reviewers in the pilot grant review process was guided by a single research question: Do the evaluations of clinical and translational research grant proposals by community members differ from those of scientific reviewers and, if so, in what ways?

## Materials and Methods

### Pilot Grant Program

This investigation was conducted in conjunction with the University of California, Irvine Institute for Clinical and Translational Sciences’ (ICTS) annual process of awarding pilot grants for clinical and translational science research projects. Each of the approximately 60 CTSAs funded by the National Institutes of Health across the country houses a component that funds pilot-phase research. The UC Irvine ICTS Pilot Awards Program supports local cutting-edge research in the early phases with the goal of nurturing these projects as they plan for larger, externally funded studies. ICTS pilot grants are designed specifically to support exceptionally innovative and/or unconventional research projects that have the potential to create or disrupt fundamental paradigms. The ICTS releases a call for proposals once each year. Awards are for 1 year of funding in the range of $25,000–$30,000. The data in this project come from the evaluation of ICTS pilot grant submissions in 2016. The project was evaluated as exempt from full review by the Institutional Review Board because it was associated with quality improvement efforts, posed no discernable risk to participants, and involved data that would be normally collected in the course of program initiatives. However, we still obtained informed consent from individuals who participated in the videotaped study sections to make them aware that interactions would possibly be used for research purposes.

Pilot grants are initially reviewed and scored by scientists or physicians (PhDs or MDs) who are affiliated with ICTS, or in some cases affiliated with other CTSAs. Proposals are assessed through an online application using a modified NIH scoring system (with additional criteria for feasibility, translational nature of the project, and likelihood of obtaining external funding), with room provided to include open-ended comments regarding the strengths and weaknesses of each proposal. Following the initial evaluation of proposals, scores and comments are reviewed and used to inform a decision as to which proposals will advance to a further round of review. The remaining highly scored proposals are then discussed in a “study section” where a panel of scientists considers each application in more depth and determines whether to alter the initial scores provided. Following the study section, determinations of pilot funding are made based on the final rankings, and the ability to meet the budget requirements of the grants with available funds.

### Community Reviewers

Community reviewers participating in this study had diverse professional backgrounds, work environments (community benefit or community-based practices), and education levels, but none were employed by the university. Although there was no expectation that community reviewers had a specific area of scientific expertise, efforts were made to match each grant application by topic, so that community reviewers would be more likely to offer feedback relevant to that domain (e.g., a community member working in children’s health might review a scientific study on pediatric obesity). In total, 22 community reviewers evaluated 42 proposals. Ten of the 53 proposals had been assigned to reviewers who agreed to review, but did not complete this review. One proposal was unable to be assigned a community reviewer due to an inability to find a suitable reviewer. Out of 40 reviewers who were approached directly and asked to review, 27 reviewers agreed. The primary reason for declining to review was a lack of time.

Reviewers were recruited for this project from 3 distinct cohorts: existing community partners actively engaged in other ICTS activities (a pool of about 113 people), new community partners referred to us through ICTS-associated faculty, and reviewers recruited through our Applied Innovation Expert-in-Residence program (see http://innovation.uci.edu/programs/experts-in-residence/). Table 1 indicates the highest level of education attained by each community reviewer. Most of the MD/PhD/RN-trained reviewers had medical practices in the community, worked at community hospitals, or led community-based service organizations at the time this study occurred. Master's-level reviewers generally directed or managed community benefit organizations or programs. We were unable to determine education level for 2 reviewers; however, each of these reviewers held leadership positions within the medical device industry or medical innovations field. Though community reviewers were not employed by the UC Irvine ICTS, or another CTSA hub, they did have some existing relationship with, and knowledge of, the ICTS.

**Table 1:** Highest level of education of community reviewers

Degree	Number of reviewers with this degree/combination
PhD	5
MD	4
MD+MBA	1
PsyD	1
RN	1
RN+PhD	1
MPH, MPA, MS, or MA	5
BS or undetermined	3

### Pilot Grant Evaluations

This investigation consisted of 3 phases that followed the standard review process for ICTS pilot grants. In 2016, the ICTS received 53 pilot grant proposals. Research proposals were not limited by topic, scope, or application and varied significantly in goals and areas of focus including developing epigenetic models of diseases, designing prosthetic body parts, or identifying biomarkers of cancer. In Phase 1, proposals were reviewed by a minimum of 2 UCI-affiliated scientific reviewers, with 38 of 53 receiving scores from 3 reviewers. In addition to these reviewers, 48 proposals received scores from scientists at other CTSA institutions. Forty-two of the 53 proposals received scores from community or industry reviewers. Each reviewer was provided a document with guidelines for scoring and completed their evaluations of the proposals online. Community reviewers were provided a 1-hour group consultative training by phone where reviewing guidelines, methods, and context were presented and discussed, and materials disseminated to participants offering criteria to consult when reviewing proposals. Three reviewers contacted the trainers mid-review for questions related to scoring and were reminded of the criteria provided.

In addition to scoring the proposals, reviewers provided information about their own position and history of funding, and answered 2 additional questions for each proposal: (a) Taking into consideration your overall abilities as a reviewer, we would like you to please rate your level of confidence that this specific review will be helpful for others in evaluating this proposal (e.g., how sure are you about this review)? And (b) Please rate the extent to which you feel you are knowledgeable in the scientific and technical aspects of this proposal? Ratings were made on a 5-point scale (1=Extremely confident/knowledgeable; 5=Not at all confident/knowledgeable).

For each proposal, an overall impact average was computed using the scores from only the scientific reviewers, and this measure was used to determine which proposals would move on from Phase 1 of review to Phase 2. A natural cut-point was identified based on the distribution of scores and the a priori specification that no more than 20 proposals would advance to Phase 2 of the review process. The top 19 proposals, based on the evaluations of the scientific reviewers (those of both UCI and non-UCI-affiliated), advanced to study section. In Phase 2 of the investigation, 2 study sections were convened to further discuss and score the proposals that advanced. A reviewer for each study from Phase 1 represented each proposal in discussion (in 7 cases no representative was present at the study section and the chair facilitated the interaction). Study section A consisted of 7 UCI-affiliated reviewers, and study section B consisted of 4 UCI affiliates and 4 community reviewers (an additional 2 community reviewers attended by phone). Study section A reviewed 12 proposals (8 unique), and study section B reviewed 11 proposals (7 unique), with 4 proposals reviewed by both sections to allow for comparison across the groups. Study sections were chaired by NIH-funded investigators with experience sitting on study sections. Study sections were videotaped and transcribed to allow for analysis of the interactions.

Phase 3 of the study consisted of a survey distributed after the completed evaluation of grants to the community reviewers who participated in the review process. This survey was designed as an evaluative tool both to investigate how community reviewers perceived the process of grant evaluation, and to elicit suggestions to improve the process. The survey responses indicated the overall satisfaction of community reviewers and their motivations for participating in the review process.

### Data Analysis

Analyses were conducted using data from each phase of the review process. We addressed the research question in 2 ways. First we examined the overall scores for the proposals and answered the fundamental question of whether the inclusion of community reviewers in the process would alter the specific proposals that advanced from Phase 1 to Phase 2. We also compared the results for each subcategory of scoring between groups to determine if any meaningful relationships existed in the evaluations. Specifically, rank-order correlations were used to assess the strength of the associations between the evaluations of scientific reviewers and community reviewers. This was done for both the overall scores and each specific category.

Second, open-ended responses regarding the strengths and weaknesses of the proposals were coded regarding the nature of the topics discussed. Both structured and unstructured (or open) coding was used to ensure comparable data and produce a categorization grounded in and reflective of the data. The first cycle of coding was open in that topics were added to the coding list as they emerged in the data, and individual responses could contain multiple topics. This was designed to adopt an emic approach to assess the evaluative criteria, and capture strengths and weaknesses noted by participants that spanned beyond the specific labels of the scoring format. The first author engaged in the open coding effort and then shared results with the other authors. As a group, the researchers compared the emergent coding categories to the 8 specific evaluative categories given to the reviewers, and when appropriate emergent codes were collapsed into these categories to maximize consistency. Categories were established based only on consensus among the researchers. This process produced one coding category beyond the 8 evaluative criteria: quality of writing and presentation. Finally, a matrix was created including each individual review and the 9 topics and an additional round of coding was conducted by the first author, resulting in a representation of the frequency of each topic listed as a strength or weakness across the groups of reviewers.

In addition to the analysis of the specific evaluations and evaluative criteria used to assess the proposals, we also utilized the video and transcripts of the study sections to analyze interactions related to the 4 proposals that were discussed in both meetings. This included noting the number and type of participants, and nature of the discussion. In addition, we examined whether the discussion resulted in the altering of scores. This material was not coded in the same manner as the comments in the proposals; we focused specifically on the discussion of the proposals that were evaluated in both groups. Although the intent was to provide a structured analysis of communicative behaviors in these discussions, the lack of consistency in the nature and patterns of talk meant comparisons would lack reliability. Therefore, the discussions of these 4 proposals are presented as comparative case studies and analysis is confined to topics of discussion present across both sections, with specific attention given to reasons offered to alter or retain initial evaluations provided.

## Results

### Differences in Evaluations

The initial analysis consisted of examining whether the inclusion of community members’ overall scores would have altered the rankings of which proposals advanced to Phase 2. The findings revealed that, of the 19 proposals that advanced to Phase 2, 4 would have been different if the community evaluations had been included (see Table 2). However, of note is that the top 14 proposals based on the scientific reviewers were all ranked in the top 19 in the scientific/nonscientific composite score, potentially indicating something distinct among the top-ranked proposals.

**Table 2:** Ranking of proposals by overall score in Phase 1 of review

Phase 1 ranking of top grants by overall score	Proposal number ordered by scores of scientific reviewers only	Proposal number ordered by scores including community reviewers
1	31	31
2	26	26
3	2	2
4	9	29
5	35	21
6	12	35
7	46	37
8	21	9
9	48	48
10	29	12
11	37	15*
12	30	28*
13	25	30
14	41	39*
15	47*	46
16	5*	16
17	16	25
18	24*	34*
19	6*	41

* This proposal would not have been ranked in the top 19 in the other group.

We then compared the groups’ respective scoring of proposals in each individual category. Table 3 indicates the descriptive statistics regarding the scores of the scientific reviewers and community reviewers across categories, as well as the rank-order correlations between the scores of community reviewers and the scientific reviewers for each respective proposal across all categories. The analysis revealed a significant positive relationship between the scoring of the scientific reviewers and community reviewers in the categories of Overall Impact, Strength of Research Design, and Likelihood of Generating Funding. For Overall Impact the difference in scores across the 2 groups averaged only 0.07.

**Table 3:** Differences between scoring of proposals among scientific and community reviewers

Scoring category	Type of reviewers	Mean	n	SD	Rank-order correlation (*r*_*s*_)	Significance (2-tailed)
Significance and impact of research	Scientific reviewers	2.97	39	0.93	0.12	0.47
	Community reviewers	3.26	39	1.43		
Qualifications of PI and team	Scientific reviewers	2.21	39	1.05	0.23	0.18
	Community reviewers	2.59	39	1.48		
Innovation	Scientific reviewers	2.78	39	1.07	0.20	0.21
	Community reviewers	3.26	39	1.19		
Scientific strength of research design	Scientific reviewers	3.55	37	1.29	0.34	0.04**
	Community reviewers	3.32	37	1.53		
Scientific environment	Scientific reviewers	2.23	39	1.08	−0.05	0.78
	Community reviewers	2.85	39	1.46		
Translational nature	Scientific reviewers	2.68	39	1.12	0.17	0.29
	Community reviewers	3.21	39	1.53		
Project feasibility	Scientific reviewers	2.97	39	0.95	0.18	0.26
	Community reviewers	3.31	39	1.44		
Likelihood of generating funding	Scientific reviewers	3.24	38	1.22	0.43	0.01**
	Community reviewers	3.47	38	1.59		
Overall impact	Scientific reviewers	3.21	39	1.09	0.28	0.09*
	Community reviewers	3.28	39	1.54		

**p<0.05; *p<0.1.

### Comments on Reviews

**Fig. 1:**
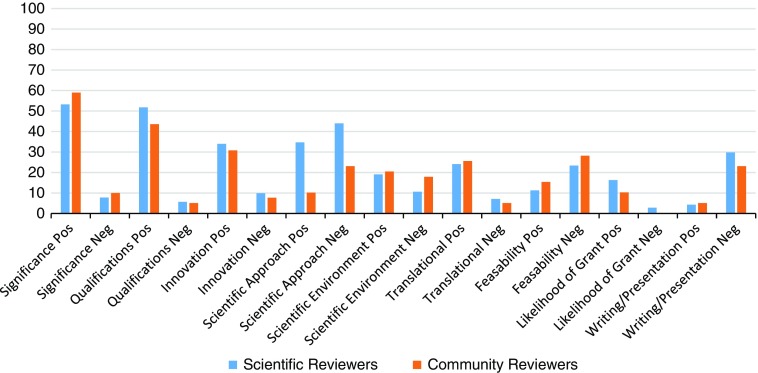
Percentage of reviewers that mentioned a category in the strengths or weaknesses of a grant evaluation. Pos, positive comment; Neg, negative comment.

Although scientific reviewers and community reviewers showed multiple areas of similarity in the groups’ respective quantitative scoring of grant applications, the open-ended comments on the strengths and weaknesses of the proposals revealed one substantial divide in the focus of reviews; specifically, the evaluation of the scientific approach. Scientific reviewers were more likely to comment upon aspects of a proposal related to the significance of the work, qualifications of the researchers, and scientific approach, and conversely community reviewers were less likely to mention these categories in evaluations. Fig. 1 indicates the percentage of reviews that mentioned each respective category.

Also of note is the frequency with which individuals noted the writing or presentation of a grant as a weakness. This comment most often was related to a lack of clarity, but also involved typographical errors, use of vague language, or poor organization.

### Case Studies of Proposals

To add additional insight into the potential role of community reviewers in the grant evaluation process we examined the discussions that took place in the 2 study sections convened to discuss proposals that had advanced past the first scoring stage. For each of the 4 proposals that were discussed across both study sections, we identified a common topic of conversation that came up across both study sections (respective topics are listed in the first column of Table 4). Because the panels had access to the strengths and weaknesses listed by the original slate of reviewers, it is unsurprising that the discussants focused on similar issues across the 2 study sections. However, the comparison of cases revealed that despite starting with the same scores and comments from reviewers, the strengths and weaknesses were characterized differently across the 2 study sections, and this discrepancy resulted in different scoring decisions in each case. Table 4 displays the comparative cases, an example quote representing the discussion in the respective section, and the scoring decision.

**Table 4:** Comparison of discussion of identical proposals across different study sections

	Shared topic of discussion		Exemplar quote	Section disposition
Proposal 1	Explanation of the proposed connection between genotype and phenotype	Study Section A	Speaker 1: “That’s a bit of leap, I think.” Speaker 2: “Yeah, I think you’re right. Maybe it makes sense, but if they don’t cite those connections, then you’re right. It looks more like a fishing expedition that they’re targeting.”	Scores moved down
		Study Section B	“And there are other minor weaknesses related to quantitation that’s small that’s in the correlation of the genotype - the parameters used to correlate genotype/phenotype.”	Scores held same
Proposal 2	Presence of significant amount of pilot work completed	Study Section A	“It’s hard to imagine in this day and age having too much preliminary data. It’s so conservative now they almost want everything all done.“	Scores held same
		Study Section B	“So they’ve actually done this work in a small number of animals already and it’s not clear since this is pilot funding that they’re asking for. It’s not clear why additional pilot funding is needed to do another pilot when they’ve already piloted it.”	Scores moved down
Proposal 3	Concern about the appropriateness of the control group used	Study Section A	“All three reviewers had issues with this control group and they were looking at different types of samples.”	Scores held same
		Study Section B	“That they didn’t put a rationale for why they didn’t look at the same type of sample and why they think or why they proposed that as a reasonable control group. And I think that’s sort of the big problem with the study.”	Scores moved down
Proposal 4	Vague methodology	Study Section A	“Let’s see, I think methodology is very important. When I first read this, I wasn’t very familiar with the area and there are some things that I thought weren’t explained well and I thought it was my own fault, not for being aware of the area. But when I read it in more detail last night, and the way they present this methodology, that’s sort of a conventional approach. A lot of questions arise.”	Scores held same
		Study Section B	“So I also thought it wasn’t – my sense is there wasn’t a very well written application. There’s a lot of kind of clichés in the introduction to this thing. I think it’s a little bit overstated.”	Scores moved down

Study Section A was comprised of scientific reviewers only. Study Section B included community reviewers as well.

When discussing proposal 1, the reviewers noted confusion about one of the scientific arguments related to the connection between the genotype present and the phenotype that emerges. In study section A, this lack of clarity was characterized as a potential “fishing expedition” and resulted in scores below those of the initial reviewers; however, in study section B the incomplete argument was seen as a minor weakness and no revised scores were outside the initial range. The difference in framing and consequences is even more stark in the evaluation of proposal 2, which was characterized by the presence of a significant amount of existing pilot data. In study section A, the large amount of pilot data was viewed as a significant strength that might aid in securing of future funding, but in study section B, the pilot data were interpreted to mean that the application might not be appropriate for this type of grant. In the evaluations of proposals 3 and 4, there was no apparent difference in the framing of the strengths and weaknesses, but in each case one section determined there was cause to move the scores outside the range of the initial reviewers, and the other section determined the scores should stay in the initial range. In 3 out of the 4 cases, then, the study section with the community reviewers downgraded the scores after the discussion. In each of these cases, comments regarding altering the scores were voiced only by scientific reviewers.

### Experience of Community Reviewers

A survey of community reviewers administered after the study sections had concluded assessed the opinions individuals had regarding their involvement in evaluating pilot grants. Table 5 indicates the average responses provided by the community reviewers (n=19). The survey results indicated that being able to give back and support developing scientists was among the strongest motivations for participating in the review process, whereas the desire to influence the decision to fund particular topics was one of the weakest motivations. Additionally, when asked to list their overall satisfaction with the review process on a scale of 0–100 (with 0 being not satisfied at all, and 100 being completely satisfied), the mean score was 87.16, indicating a very high level of satisfaction.

**Table 5:** Motivations expressed by community reviewers regarding reasons for participating in the peer review process (1=disagree strongly; 5=agree strongly)

So that I could be an active participant in a research community	4.16
Because I wanted to influence the decision to fund particular topics	3.37
Because I was interested in learning about the cutting-edge science	4
Because I was curious about what was going on at UCI	3.74
To be able to give back, and support developing scientists	4.53

## Discussion

Regarding the research question of whether the evaluations of clinical and translational research grant proposals by community members differ from those of scientific reviewers the findings are mixed. The ratings between the 2 groups did differ slightly, but in a manner that would have had only a minor influence on the progression of proposals through the review process. Additionally, significant correlations in scoring were found in a few categories, but not all. Also of note is that among the 4 proposals reviewed by both the study section solely of scientific reviewers and the study section including community reviewers, the sections were consistent in not ranking any of these 4 proposals high enough to eventually receive funding. Quantitatively, there is evidence that the inclusion of community reviewers in the peer review process does not significantly alter rankings.

The similarities in scoring of proposals occurred despite apparent differences in the nature of perceived strengths and weaknesses of the proposals. As might be expected, scientific reviewers commented extensively on the relative significance of the work and the study design. Although community reviewers were given the freedom to comment on any aspects of the proposals, they confined feedback to composition of the project and its presentation.

Taken together, these findings have 2 significant implications regarding the potential role of community reviewers. First, the small potential influence of the community reviewers suggests that the inclusion of these individuals would not have significantly altered the application of evaluative standards, or detracted from the scientific rigor applied to assessments. In the study section involving the community reviewers there was no evidence that discussion of the scientific merits of the proposals were limited or constrained in any way.

A second implication of these findings is that community reviewers may provide a distinct yet valuable form of knowledge that aids in the evaluation of grant proposals. One way of interpreting the focus of community reviewers is that they based evaluations on aspects of the proposal they felt comfortable judging. Whereas the scientific reviewers evaluated the proposals in terms of what they considered expert science, the community members took a more abstract view of what constituted an expert grant proposal—distinct categorizations that often, but do not always converge. As working professionals with experience communicating about projects, community reviewers were capable of evaluating whether a proposal was clear and convincing in its presentation. Given that a central goal of these projects was to facilitate ongoing funding through future grants, consideration of more stylistic or management-oriented aspects of studies at this stage may serve as a solid indicator of the success of future proposals. The traditional idea of peer review of scientific proposals rests on the principle that scientists immersed in a similar domain of practice related to a proposal have the requisite expertise to discern the relative quality and promise of research, and in turn that others lack similar expertise. However, in instances of peer review, what is being evaluated is not the actual expertise of the researcher as a scientist—the actual practice of science—but rather the communication of that expertise in the form of a proposal. As the reviews and discussion of proposals revealed, there were several factors related to the evaluation of the grant proposals that were beyond questions of scientific or technical merit, including the writing of the proposal (or application) itself. The inclusion of community reviewers creates a “hybrid forum” of expertise [18] in the evaluation process that broadens the diversity of expertise applied to the evaluation. This diversity of expertise may be helpful in avoiding the myopia associated with specialist expertise, while retaining the distinct expertise that scientists provide. Previous approaches to integrating community members into the review of pilot grants developed scoring guidelines for community members that were distinct from those of scientific reviewers and focused on aspects of the community-academic partnership [19]. The findings of this study indicates separate scoring systems for the respective groups may not be necessary, and there may be relational and communicative benefits to integrating the work of scientific and community reviewers.

Finally, these findings provide evidence of the need to further study the process through which clinical and translational science proposals are evaluated. Analysis of the discussions in the study sections showed that individuals with access to identical review scores and comments can interpret those results in distinctly different ways. Future work should assess ways to establish greater standardization of approaches to evaluating grant proposals, training in facilitation of study sections, and ongoing process improvement in the administration of CTSA funds.

### Implications for Community Engagement

The extremely high levels of satisfaction and enthusiasm expressed by community reviewers related to participation in the evaluative process demonstrates potential benefits for engaging community members at the incubation stage of research efforts. Engagement of community members at the initial stages of designing and evaluating projects recognizes translational science as a cycle of evolution whereby each stage influences the next. Confining community engagement efforts to later stage translational science efforts may risk overlooking ways community members might shape the presentation of work or help select the most promising initiatives. Furthermore, both the scoring of proposals by community reviewers and their espoused motivations for participating in the review process suggests that any concerns about nonscientific reviewers undermining or tainting assessments of scientific merit may be misguided. In survey responses community reviewers expressed relatively low interest in actively influencing proposal decisions, and this is consistent with the earlier finding that the comments community reviewers made were not focused on scientific aspects of the proposals.

At a minimum, the engagement of community members in the review process may have significant symbolic and relational value for those participating in the evaluation process. To recruit reviewers, UCI’s ICTS utilized a network of community members that already had some relationship with the institution, its affiliates, or its initiatives. Positive participation in the review process served as a way to deepen their relationship with the ICTS. Moving forward, these individuals can serve as potential advocates for the ICTS and its programs.

### Limitations

There are several aspects of this investigation that limit the generalizability of the findings. Of greatest concern is the scope and context of the study, and the nature of the participants. This research examines proposals from a single site, one round of applications, and a limited number of reviewers that were inconsistently distributed across proposals. Additionally, although our community reviewers were not working scientists and not involved in applying for or evaluating grants, they predominantly had advanced degrees and all had some existing relationship with the ICTS. The educational and professional experience of these participants restricts the generalizability of these findings to other, potentially more diverse, groups of community stakeholders that may possess less familiarity with scientific or medical contexts. We were able to validate that compared with the community participants the scientific reviewers perceived themselves as significantly more knowledgeable in the scientific and technical aspects of the proposals evaluated by both groups when; with a significant difference in the scores on this survey question [M=−0.34, SD=0.96, CI (−0.65, −0.02); *t* (37)=−2.18, *p*=0.036]. However, this difference is not large in absolute terms, and it is certainly possible the results presented are unique to UCI’s ICTS or to the specific group of community reviewers who participated. Moving forward, it is critical to study the inclusion of a greater diversity of community members in the research evaluation process.

Another limitation is that a clear majority of the proposals evaluated were focused on the early stages of translational science (40 of 53 applications were stages T0–T1), meaning we were unable to assess whether differences existed between scientific and community reviewers based on the translational stage of the research. This restricts our ability to examine the nature of the expertise or insights that community members might add to the evaluation process, as it is possible community members may add additional knowledge at later stages of translational research that involve issues such as publicly disseminating and applying results. Additional research is needed to determine whether community reviewers may play a different or distinct role in the evaluation of proposals further down the translational spectrum. Another challenge is that the nature of the pilot grant program means that there is tremendous diversity in the topical nature of research proposed. Future work should evaluate whether results differ across specialized fields.

Finally, though the survey results indicate that the community reviewers were largely satisfied with the process of participating in grant evaluations, we do not have evidence regarding any other outcomes this effort may have for ongoing efforts at community engagement. Specifically, future research is needed to assess whether involving community members earlier in the research process may improve relationships between community members and scientists. The results of this research provide a foundation for longitudinal work that examines community-scientist relationships across the research lifecycle.

### Conclusion

In looking together at evidence from the community reviewers’ ratings of proposals, participation in the study sections, and their respective reflections on participation, what emerges is a picture of community reviewers as enthusiastic and engaged, yet largely restrained in their efforts to influence evaluations. Overall, this investigation demonstrated the feasibility of engaging community reviewers in the evaluation of ICTS pilot grants and indicated that inclusion of community members would have minimal influence on outcomes of proposal evaluations. Though additional work is needed to more rigorously assess the specific outcomes related to the participation of community members in reviews, the results of this work serve as a foundation to examine if reviewing is a potential avenue to bolster community engagement efforts.
